# A glycogen storage disease type 1a patient with type 2 diabetes

**DOI:** 10.1186/s12920-022-01344-3

**Published:** 2022-09-27

**Authors:** Yi Sun, Wenhui Qiang, Runze Wu, Tong Yin, Jie Yuan, Jin Yuan, Yunjuan Gu

**Affiliations:** 1 Department of Endocrinology and Metabolism, Affiliated Hospital of Nantong University, Nantong, Jiangsu China; 2 Medical College, Nantong University, Nantong, Jiangsu China; 3 Department of Endocrinology, Affiliated Hospital of Nantong University, 20 Xisi Road, Nantong, 226001 Jiangsu China

**Keywords:** GSD1a, T2DM, G6PC gene

## Abstract

**Background:**

Glycogen storage disease type 1a (GSD1a) is an inborn genetic disease caused by glucose-6-phosphatase-α (G6Pase-α) deficiency and is often observed to lead to endogenous glucose production disorders manifesting as hypoglycemia, hyperuricemia, hyperlipidemia, lactic acidemia, hepatomegaly, and nephromegaly. The development of GSD1a with diabetes is relatively rare, and the underlying pathogenesis remains unclear.

**Case presentation:**

Here we describe a case of a 25-year-old Chinese female patient with GSD1a, who developed uncontrolled type 2 diabetes mellitus (T2DM) as a young adult. The patient was diagnosed with GSD1a disease at the age of 10 and was subsequently treated with an uncooked cornstarch diet. Recently, the patient was treated in our hospital for vomiting and electrolyte imbalance and was subsequently diagnosed with T2DM. Owing to the impaired secretory function of the patient’s pancreatic islets, liver dysfunction, hypothyroidism, severe hyperlipidemia, and huge hepatic adenoma, we adopted diet control, insulin therapy, and hepatic adenoma resection to alleviate this situation. The WES discovered compound heterozygous mutations at the exon 5 of G6PC gene at 17th chromosome in the patient, c.648G>T (p.L216 L, NM_000151.4, rs80356484) in her father and c.674T>C (p.L225 P, NM_000151.4, rs1555560128) in her mother. c.648G>T is a well-known splice-site mutation, which causes CTG changing to CTT at protein 216 and creates a new splicing site 91 bp downstream of the authentic splice site, though both codons encode leucine. c.674T>C is a known missense mutation that causes TGC to become CGC at protein 225, thereby changing from coding for leucine to coding for proline.

**Conclusion:**

We report a rare case of GSD1a with T2DM. On the basis of the pathogenesis of GSD1a, we recommend attentiveness to possible development of fasting hypoglycemia caused by GSD and postprandial hyperglycemia from diabetes. As the disease is better identified and treated, and as patients with GSD live longer, this challenge may appear more frequently. Therefore, it is necessary to have a deeper and more comprehensive understanding of the pathophysiology of the disease and explore suitable treatment options.

**Supplementary Information:**

The online version contains supplementary material available at 10.1186/s12920-022-01344-3.

## Background

Glycogen storage disorders (GSDs) are a group of inborn metabolic errors caused by various genetic defects in glycogenolytic/synthetic enzymes or protein mutations that regulate glycogen metabolism, accompanied by abnormalities in glycogen store or use [1]. Different types of GSD are classified according to the deficient enzymes and affected tissues. GSD1 is usually divided into two subtypes [2], namely, GSD1a and GSD1b. The subtype GSD1a is caused by a deficiency in glucose-6-phosphatase-α (G6Pase-α), whereas the subtype GSD1b results from the absence of the glucose-6-phosphate (G6P) transporter (G6PT). Blood glucose homeostasis between meals is mainly maintained by the complex of G6PT and G6Pase-α, which catalyzes the hydrolysis of intracellular G6P to glucose in the terminal step of gluconeogenesis and glycogenolysis in the liver, kidney, and intestines [2]. Therefore, GSD1 can result in endogenous glucose production (EGP) disorders [3]. GSD1a is the most prevalent subtype and represents approximately 80% of GSD1 cases [1].

GSD1a is an autosomal recessive genetic disease with an incidence of about 1 in 100,000 [4]. It is caused by an inactivating mutation in the gene G6PC, which encodes G6Pase-α. The human G6PC gene is a single-copy gene composed of five exons on chromosome 17q21, which encodes the highly hydrophobic, 357-amino acid glycoprotein G6Pase-α [2]. More than 100 G6PC mutations have been reported at present. Furthermore, GSD1a occurs in various ethnic groups, among which c.648G>T (p.L216L) and c.248G>A (p.R83H) are the most common mutations in the Chinese, Japanese, and Korean populations [5, 6].

GSD1a patients typically have hypoglycemia from an impaired last step of gluconeogenesis and accompanying hyperuricemia, hyperlipidemia, and lactic acidemia from excess G6P as discussed above [7]. The accumulation of glycogen in hepatocytes and proximal renal tubules leads to hepatomegaly and nephromegaly. Hepatomegaly is further exacerbated by the accumulation of lipids in the liver leading to obvious hepatosteatosis. The consequences of further progression of the disease include growth delay, hepatic adenoma, chronic renal disease, gouty arthritis, osteoporosis, and pulmonary hypertension [8]. Diet therapy is the cornerstone of GSD1a treatment [1]. Regular intake of carbohydrates is necessary to prevent hypoglycemia and achieve good metabolic control. Cornstarch is slowly digested; thus, it can steadily release glucose between meals. The use of uncooked cornstarch in adult GSD1a patients has been proven as a simple and effective long-term treatment.

For individuals who are primarily suffering from a disease with reduced EGP, it seems paradoxical that GSD1a patients would develop diabetes. In actuality, the development of GSD1a with concomitant diabetes is very rare. Here, we describe a young woman with GSD1a and T2DM, and explore the possible pathogenesis, which may provide a basis for further understanding of the disease.

## Case presentation

### Part 1 endocrinology hospitalization

In March 2019, a 25-year-old Chinese female patient was admitted to the endocrinology department of Nantong University Affiliated Hospital with hepatosplenomegaly for more than 20 years and vomiting for 4 days. At the age of 4, the patient was examined in the hospital because of repeated epistaxis, anemia and developmental delay and was observed to have hepatosplenomegaly. At the age of 10 years, the patient underwent liver biopsy due to abdominal distension, and the results suggested GSD1a. Since then, the patient was advised to consume uncooked cornstarch as a staple food. The patient’s blood was sent to Shanghai Xinhua Hospital for genetic testing at the age of 20. Compound heterozygous mutations, namely, 727G>T(L216L) and 674T>C (L225P), were detected in exon 5 of the G6PC gene (Additional file 1: Figure S1). The patient’s father and mother were heterozygotes who were found to carry 727G>T and 674T>C, respectively. In addition, the results of other biochemical examinations showed that the patient also suffered from hypothyroidism, hyperlipidemia, and osteoporosis and received further treatment accordingly.

The patient’s parents were healthy and had no family history of consanguinity. Menarche was reported to be at 14 years old with normal succeeding afterward.

Physical examinations showed that the patient was 155 cm in height and 40 kg in weight, below the average values of the patient’s peers (BMI = 16.64 kg/m^2^). Additionally, the patient’s secondary sex characteristic had developed normally. The patient’s abdomen was distended, and the liver was firm and palpated 10 cm below the right costal margin. Additionally, the spleen was palpated 4 cm below the left costal margin with hard and blunt edges. No obvious percussion tenderness was observed over the liver region.

**Fig. 1:**
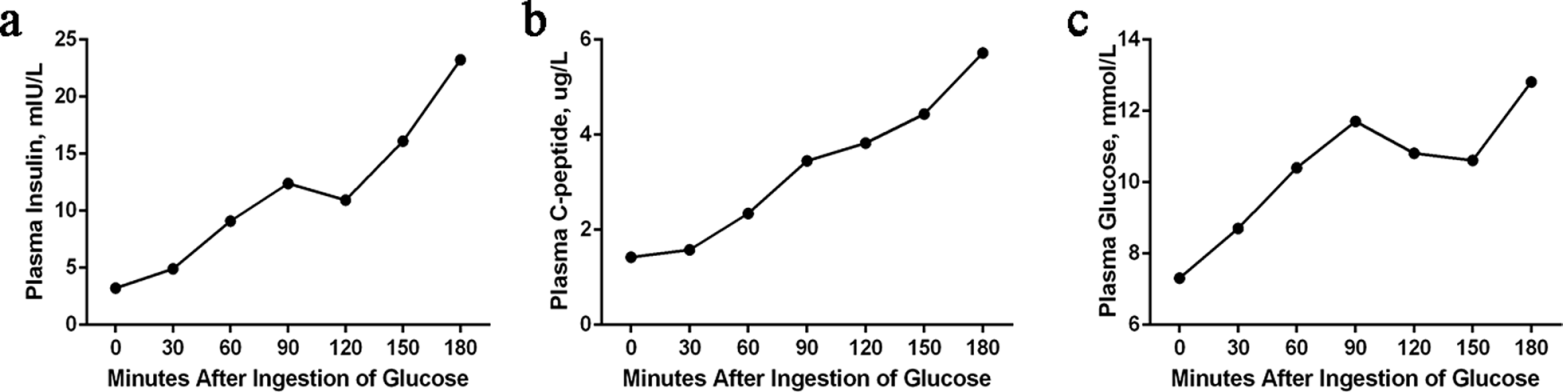
Islet function of the patient. Plasma insulin levels (**a**), C-peptide concentration (**b**) and glucose concentration (**c**) after ingestion of glucose

Laboratory examinations showed slight anemia (hemoglobin 93 g/L), increased liver enzymes (AST 50 U/L), increased serum lipid levels (TC 8.7 mmol/L, TG 15.83 mmol/L), increased serum amylase (340 U/L), and disordered serum electrolytes (K^+^ 2.9 mmol/L, Na^+^ 137 mmol/L) (Additional file 7: Table S1). The patient’s fasting plasma glucose was 11.4 mmol/L, and her HbA1c was 5.5%. Islet cell autoantibody, insulin autoantibodies (IAA), antibody against glutamate decarboxglase (GAD-Ab), tyrosine phosphatase-like IA-2 autoantibody (IA-2A), and zinc transporter 8 antibody (Znt8) were all negative. Combined with the results of the islet function test (Fig. 1a–c), the patient was diagnosed with T2DM. The patient’s thyroid function showed FT3 levels of 5.15 pmol/L, FT4 of 8.47 pmol/L, and TSH of 14.99 mIU/L.

Imaging studies demonstrated some evidence supporting hepatomegaly and space-occupying lesions. Abdominal ultrasound showed several hypoechoic space-occupying lesions, with the largest size at 88 × 76 mm, showing clear boundaries and regular shapes. Abdominal MR showed that the liver was enlarged, with a smooth capsule and multiple round-shaped space-occupying lesions. The larger lesion was located in the right posterior lobe of the liver, with an approximate size of 8.0 × 6.4 cm (Additional file 2: Figure S2 a, c, e, g).

The patient was treated with corn starch treatment. In terms of blood glucose control, we first administered a subcutaneous injection of insulin aspart before three meals (6 u before breakfast, 4 u before middle, 4 u before dinner). Capillary blood glucose monitoring showed that the blood glucose was well controlled at night, while glucose fluctuated greatly during the day (Additional file 3: Figure S3). Therefore, we opted to switch to Humula 50R (10 u before breakfast) combined with insulin aspart (4 u before dinner) to provide a better blood glucose control. Oral levothyroxine sodium was used to regulate thyroid function. Additionally, we administered other adjuvant treatments such as liver protection, lipid-lowering, and calcium supplement.

### Part 2 hepatobiliary surgery hospitalization and follow-up

**Fig. 2:**
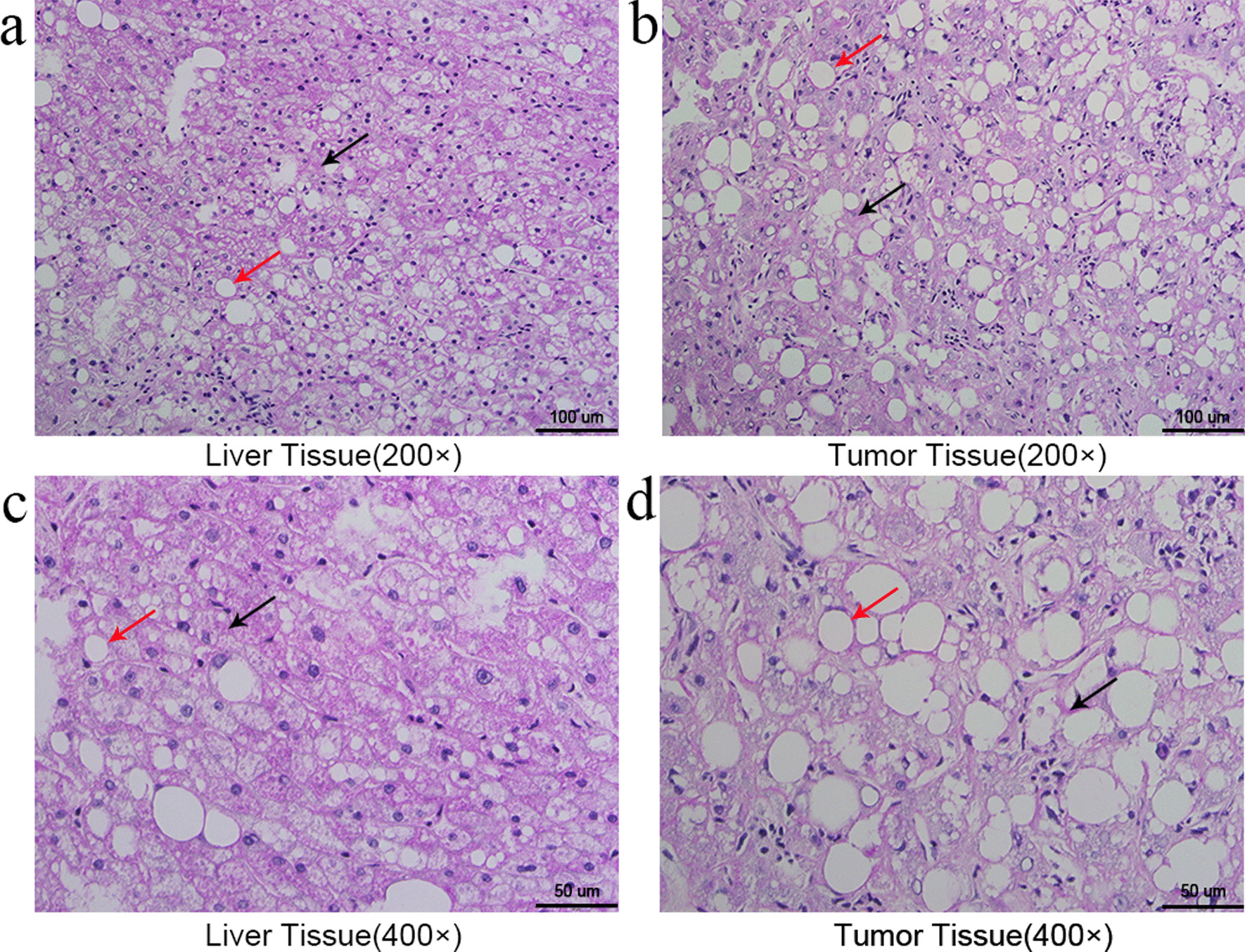
We observed PAS-stained sections using an Olympus BX41 fluorescence microscope, and image processing software cellSens was used for image acquisition and processing. PAS staining suggested liver tissue (**a**: 200 ×, **c**: 400×) had more glycogen deposition than tumor tissue (**b**: 200 ×, **d**: 400 ×). The red arrows represented lipid droplets. The black arrows represented deposition of glycogen

Upon re-examination of abdominal MR, the size and number of the space-occupying lesions were shown to have increased (Additional file 2: Figure S2 b, d, f, h). Therefore, the patient was admitted to the Department of Hepatobiliary Surgery, Affiliated Hospital of Nantong University in March 2020. The postoperative pathological results suggested hepatic adenoma and steatosis. PAS staining demonstrated increased glycogen deposition in the liver tissue than that in the tumor tissue (Fig. 2a–d).

**Fig. 3:**
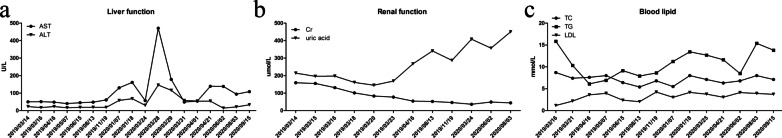
The levels of liver function (**a**), renal function (**b**) and blood lipid (**c**) during the follow-up period

After using insulin for 2 months, we discontinued insulin aspart and retained only Humula 50R (8 u before breakfast) to control blood glucose. According to the follow-up results, the patient’s blood glucose levels were well controlled (Additional file 4: Figure S4). The patient’s weight also gradually increased (Additional file 7: Table S1). However, after treatment, liver function and blood lipids were not observed to improve. Renal function examination revealed that uric acid and urinary albumin concentration also gradually increased (Fig. 3a–c, Additional file 7: Table S1).

**Fig. 4:**
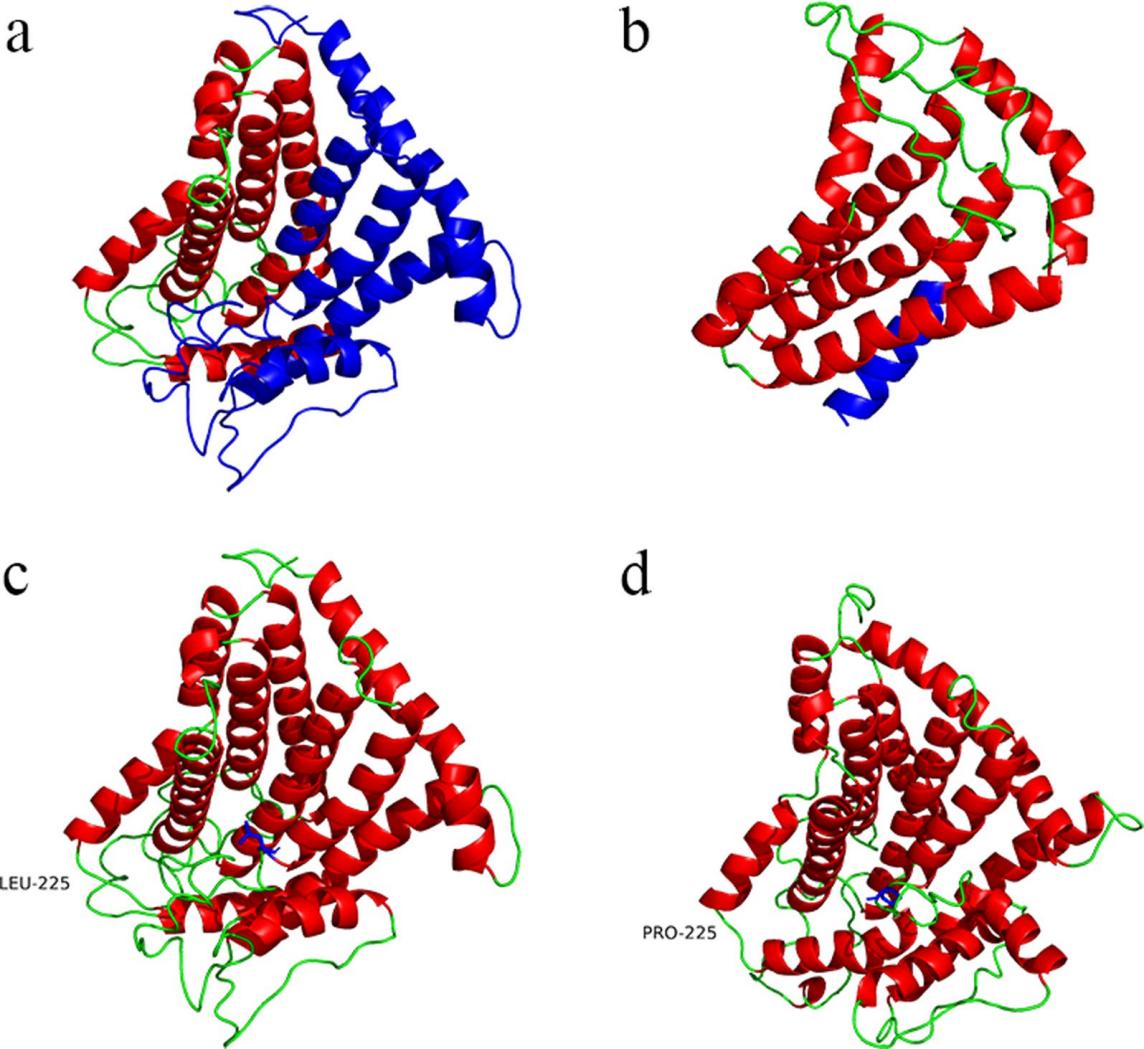
Protein structure prediction. **a** G6PC wild type, blue region is amino acid 188–357. **b** G6PC:NM_000151.4: exon5: c.648G>T: p.L216L mutant. The blue region is the frame shifted amino acids 188–201. **c** G6PC wild type, marked in blue as 225LEU. **d** G6PC:NM_000151.4: exon5: c.674T>C: p.L225P mutant, marked as 225PRO in blue

Recently, after informing the patient and family, and obtaining the signed informed consent, we collected the peripheral blood of the patient and her parents, and sequenced the entire exome of them to find the mutant gene, and then used first generation of sequencing to verify the mutation in the patient and her families. The result of exome sequencing suggests that there were heterozygous mutations on exon 5 of G6PC gene, c.648G>T (p.L216 L, NM_000151.4, rs80356484) from her father and c.674T>C (p.L225 P, NM_000151.4, rs1555560128) from her mother (Additional file 5: Figure S5, Additional file 6: Figure S6). Gene Reviews indicate that G727T is an alias for c.648G>T (https://www.ncbi.nlm.nih.gov/books/NBK1312/). c.648G>T is a well-known splice-site mutation and the most prevalent mutation (54% of the alleles) in Chinese patients [9], which causes CTG changing to CTT at protein 216 and creates a new splicing site 91 bp downstream of the authentic splice site, though both codons encode leucine [10, 11]. c.674T>C is a known missense mutation in GSD1a patients [12, 13] that causes TGC to become CGC at protein 225, thereby changing from coding for leucine to coding for proline. Furthermore, we predicted the protein structures of wild-type and two mutant G6PC proteins using I-TASSER and PyMOL software, respectively (Fig. 4a–d).

## Discussion and conclusions

Generally, patients with GSD1a have persistent episodes of fasting hypoglycemia. Cases of GSD1a combined with diabetes are reported relatively rarely. In fact, there have only been five reported cases of GSD1a with diabetes mellitus [14, 15], including one middle-aged adult case, two young adult cases, and two pediatric cases. No sex difference has been reported for the onset. The Annual Symposium of the Society for the Study of Inborn Errors of Metabolism has reported four cases of GSD1a patients with DM and pointed out that glycemic control can be achieved through the reduction of weight through strict diet control [15]. In addition, a 34-year-old South Asian obese female patient with GSD1a and DM demonstrated stable glycemia control after diet control and exercise for weight loss and administration of oral canagliflozin and acarbose [14]. In our case, the patient was an underweight adult female (BMI = 16.64 kg/m^2^) who had no obvious insulin resistance. Glycemic control was finally achieved through the use of Humula 50R combined with insulin aspart injection.

The pathogenesis of GSD1a combined with DM remains unclear. This may be for the following reasons:

First, patients with poorly controlled GSD1a often have prolonged periods of low blood glucose levels, and with the passage of time, the patient’s response to hypoglycemia gradually decreases, even to the point of developing ignorance of hypoglycemia. This adaptive mechanism may be mediated in part through the down-regulation of the glucose receptor on the β-cell membrane GLUT2 for the reduction of insulin secretion. These patients gradually develop from a transient decrease in insulin to a permanent state of insulinopenia and lose the ability to secrete appropriate amounts of insulin over time. Eventually, this leads to the development of diabetes [16, 17]. In this case, there was almost no observable episode of hypoglycemia during the course of the disease. This may be related to the patient’s long-term tolerance to hypoglycemia, which led to a decrease in insulin secretion and eventual development of diabetes.

Second, islet function in the patient demonstrated impaired insulin secretion both upon fasting and 2 h postprandially. A previous study reported insulinopenia in five adult GSD I patients [18]. Therefore, hyperglycemia seen in this disease accompanied by increasing age suggests that decreased insulin responsiveness may be a slowly developing adaptive process.

Third, this patient always had liver dysfunction. It has been reported that progressive liver dysfunction was responsible for the development of diabetes mellitus [19]. The pathogenesis of glucose intolerance caused by liver dysfunction remains unclear. This may be related to the decreased insulin sensitivity of peripheral tissues and decreased glucose utilization [20–22].

Fourth, the patient’s serum amylase was observed to have increased during hospitalization in our hospital; thus, pancreatic injury should be considered. Hirashima et al. reported a case of GSD 1a with acute pancreatitis and pointed out that severe hyperlipidemia was an important risk factor for acute pancreatitis [23]. Pancreatic injury can cause pancreatic β-cell dysfunction, which in turn leads to insulin deficiency.

Additionally, after functional identification of G6PC gene mutations, most of the mutations were observed to completely abolish G6Pase-α activity, whereas some mutations retain varying degrees of residual enzymatic activity [2]. The intake of glucose through frequent meals with carbohydrate diets with low glycemic index food and residual enzyme activity may lead to glucose intolerance and decreased peripheral insulin sensitivity through the consistent elevation of blood glucose levels [24]. However, previous studies have found that there is almost no strict genotype–phenotype relationship for G6PC mutations [25]. Although our patient has not been tested for enzyme activity, this possibility needs to be considered.

There are no current guidelines on treating hyperglycemia in patients with GSD1a. Our patient is underweight, with impaired pancreatic islet secretion and liver dysfunction. We used insulin to control the patient’s blood glucose level. Considering the pathogenic characteristics of GSD1a, it is necessary to closely monitor blood glucose and be alert for the occurrence of hypoglycemia. Additionally, we administered raw corn starch diet, liver protection, lipid-lowering, and other adjuvant treatments and regularly followed up the blood glucose levels and related biochemical indicators.

GSD1a is a genetic disease caused by gene mutations. Lee et al. found that AAV-GPE-mediated gene therapy can correct hepatic G6Pase-α deficiency in G6pc−/− mice and prevent chronic hepatocellular adenoma (HCA) [20]. AAV-GPE is an adeno-associated virus (AAV) vector expressing G6Pase-α directed by the human G6PC promoter/enhancer (GPE). However, despite promising results of gene therapies in animal models, further investigations and refinements are required. With better recognition and treatment of the disease, the life span of GSD1a patients can be expected to be prolonged.

GSD1a combined with T2DM is relatively rarely seen, and few reports exist in the current literature. Our case reviewed and discussed the possible mechanism of the development of GSD1a with T2DM. We used insulin to control blood glucose and administered raw corn starch diet, liver protection, lipid-lowering, and other adjuvant treatments and regularly followed up the blood glucose levels and related biochemical indicators to prevent the occurrence of hypoglycemia. Currently, there is no gene therapy for clinical application, and only symptomatic treatment can be implemented. Early genetic testing and early intervention are key to extending the life of patients. As the disease is better identified and treated and the life span of GSD-1a patients is extended, this challenge may appear more frequently. Therefore, it is necessary to have a deeper and more comprehensive understanding of the pathophysiology of the disease and further explore suitable treatment options.

## Supplementary Information

**Additional file 1: Figure S1.** The mutations of G6PC found in this patient. The two mutations, shown by the black arrows, were c.727G>T, encoding p.L216L and c.674T>C, encoding p.L225P [13].

**Additional file 2: Figure S2.** Upper abdomen MRI showed an increase in the size and number of space-occupying lesions. Arrows showed the space-occupying lesion. (a, c, e, g) MRI examined on base line (2019.03.15 at presentation), the max size of lesions was about 8.0 × 6.4 cm. (b, d, f, h) MRI examined 12 months later, the max size of lesions was about 10.4 × 8.4 cm.

**Additional file 3: Figure S3** Capillary blood glucose monitoring during hospitalization in the Department of Endocrinology in March 2019.

**Additional file 4: Figure S4.** The levels of fasting plasma glucose during the follow-up period.

**Additional file 5: Figure S5.** The genotypes of G6PC gene for family members. Roman numerals indicate generations and Arabic numbers indicate individuals. Squares = males, circles = females. Unblackened and blackened symbols represent the normal haplotype and the mutant haplotype, respectively. The index patient is indicated by an arrow. The two mutations were inherited from father and mother respectively.

**Additional file 6: Figure S6.** Validation for the c.648G>T(M1) and c.674T>C(M2) of exon 5 by Sanger Sequencing.

**Additional file 7: Supplement table 1.** Examination data on presentation and follow-up.

## Data Availability

All data used during this study are included in this published article. We have uploaded the raw data to https://bigd.big.ac.cn/gsa-human/browse/HRA002095.

## Abbreviations

GSD: Glycogen storage disease type
G6Pase-α: Glucose-6-phosphatase-α
T2DM: Type 2 diabetes mellitus
G6PT: Glucose-6-phosphate transporter
EGP: Endogenous glucose production
HCA: Hepatocellular adenoma
AAV: Adeno-associated virus
GPE: G6PC promoter/enhancer
