# Supplementary figures and images for: A glycogen storage disease type 1a patient with type 2 diabetes
Source: BMC Med Genomics. 2022 Sep 27;15:205. doi: 10.1186/s12920-022-01344-3 (PMC9516787; doi:10.1186/s12920-022-01344-3)

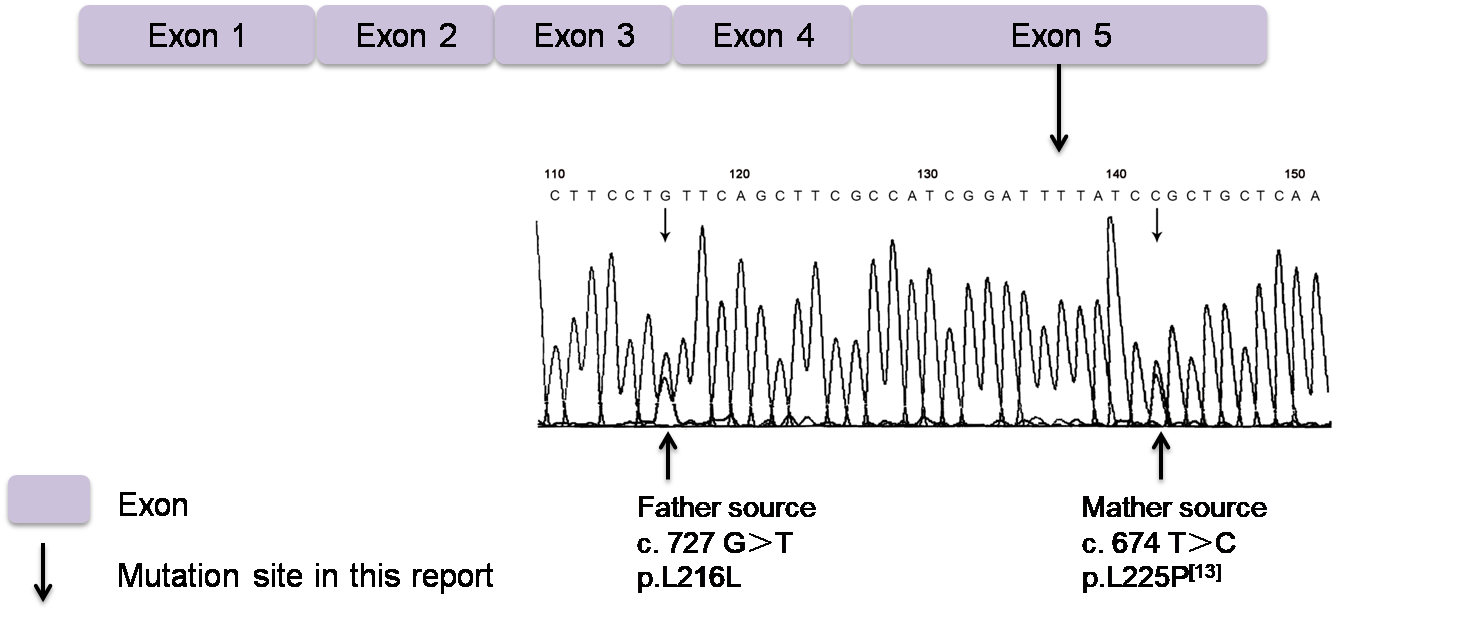

Supplement: Supplementary file 1 — Additional file 1: Figure S1. The mutations of G6PC found in this patient. The two mutations, shown by the black arrows, were c.727G>T, encoding p.L216L and c.674T>C, encoding p.L225P [13]. [file 12920_2022_1344_MOESM1_ESM.tif]

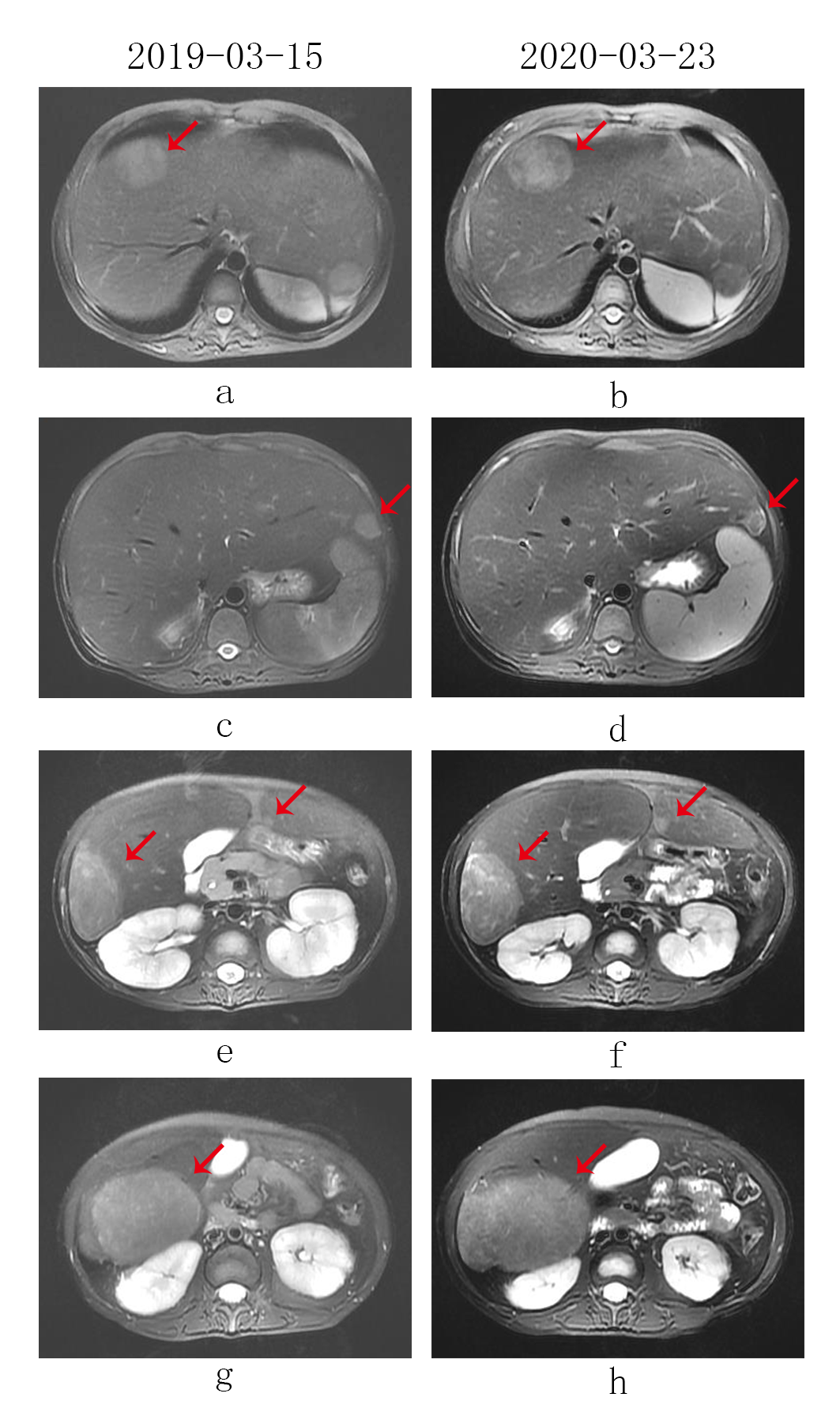

Supplement: Supplementary file 2 — Additional file 2: Figure S2. Upper abdomen MRI showed an increase in the size and number of space-occupying lesions. Arrows showed the space-occupying lesion. (a, c, e, g) MRI examined on base line (2019.03.15 at presentation), the max size of lesions was about 8.0 × 6.4 cm. (b, d, f, h) MRI examined 12 months later, the max size of lesions was about 10.4 × 8.4 cm. [file 12920_2022_1344_MOESM2_ESM.tif]

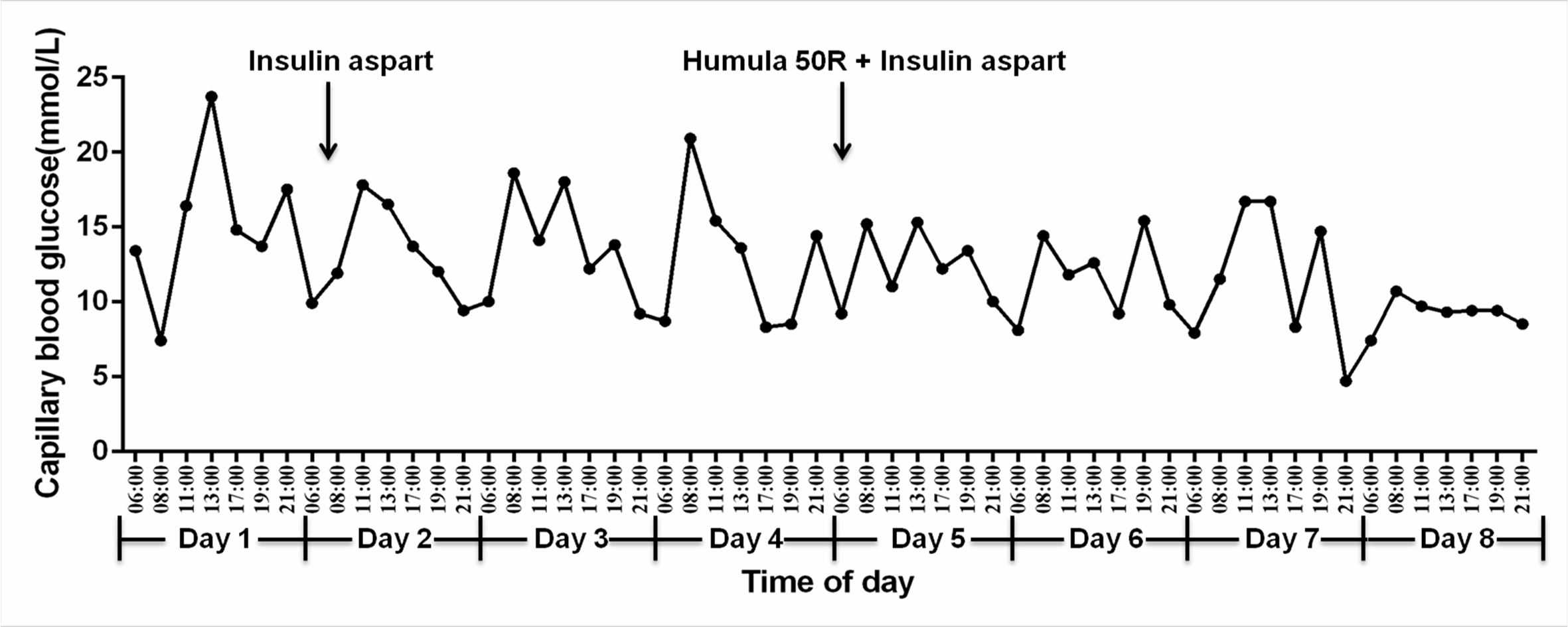

Supplement: Supplementary file 3 — Additional file 3: Figure S3 Capillary blood glucose monitoring during hospitalization in the Department of Endocrinology in March 2019. [file 12920_2022_1344_MOESM3_ESM.tif]

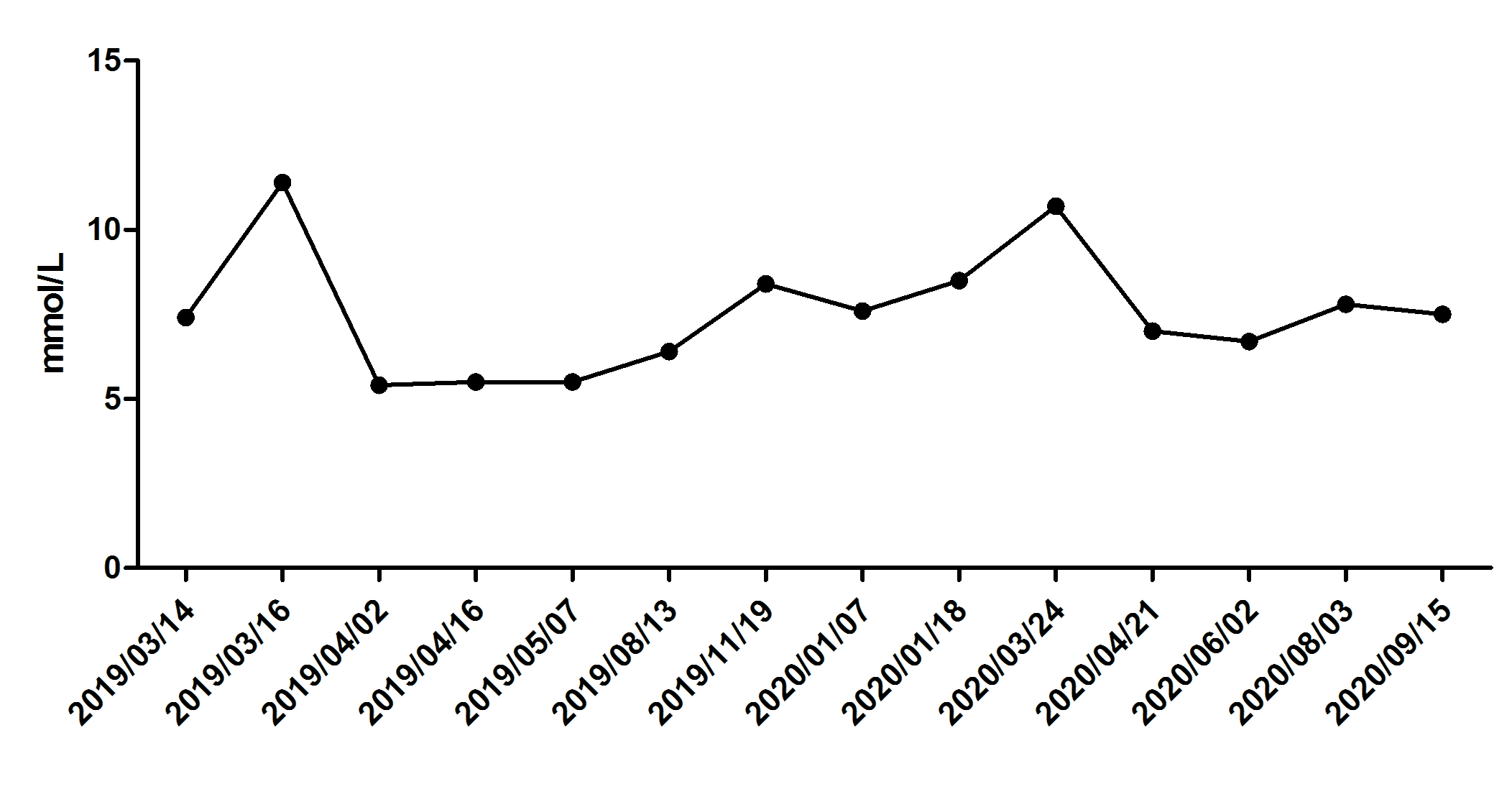

Supplement: Supplementary file 4 — Additional file 4: Figure S4. The levels of fasting plasma glucose during the follow-up period. [file 12920_2022_1344_MOESM4_ESM.tif]

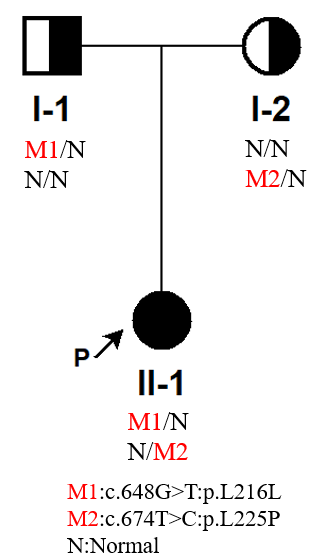

Supplement: Supplementary file 5 — Additional file 5: Figure S5. The genotypes of G6PC gene for family members. Roman numerals indicate generations and Arabic numbers indicate individuals. Squares = males, circles = females. Unblackened and blackened symbols represent the normal haplotype and the mutant haplotype, respectively. The index patient is indicated by an arrow. The two mutations were inherited from father and mother respectively. [file 12920_2022_1344_MOESM5_ESM.tif]

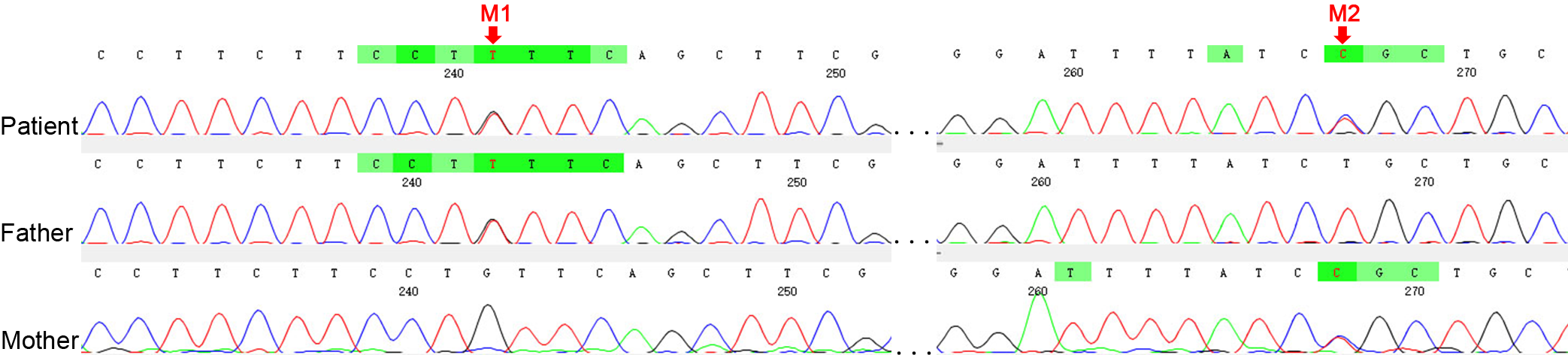

Supplement: Supplementary file 6 — Additional file 6: Figure S6. Validation for the c.648G>T(M1) and c.674T>C(M2) of exon 5 by Sanger Sequencing. [file 12920_2022_1344_MOESM6_ESM.tif]
