# Supplementary material for: A glycogen storage disease type 1a patient with type 2 diabetes
Source: BMC Med Genomics. 2022 Sep 27;15:205. doi: 10.1186/s12920-022-01344-3 (PMC9516787; doi:10.1186/s12920-022-01344-3)
Supplement: Supplementary file 7 — Additional file 7: Supplement table 1. Examination data on presentation and follow-up. [file 12920_2022_1344_MOESM7_ESM.pdf]

Table 1 Examination data on presentation and follow-up

Variable	Reference range	Baseline	9 Months	12Months	18Months
Height (cm)	-	155	-	155	155
Weight (kg)	-	40	-	43	45
BMI(kg/m2)	-	16.64	-	17.9	18.73
Hb(g/L)	110-150	93	115	109	128
ALT(U/L)	0-35	23	58	30	33
AST(U/L)	0-35	50	130	57	108
TC (mmol/l)	3.0-5.7	8.7	8	7.1	7.1
TG (mmol/l)	0-1.7	15.83	13.43	12.68	13.81
LDL (mmol/l)	1.89-4.21	1.17	4.16	3.76	3.72
FPG (mmol/L)	3.9-6.0	7.4	7.6	7	7.5
HbA1c(%)	4.0-6.2	5.5	6	6.8	6.4
Cr (μmol/l)	46-92	159	46	36	44
Ua (μmol/l)	149-369	214	287	408	450
Urinary albumin(mg/L)	＜19	1032.6	-	2246.7	13632.8
UACR(mg/mmolCr)	＜2.5	117.34	-	255.31	1549.18
K^+ (mmol/L)	3.5-5.5	2.9	3.8	3.4	4
Na^+ (mmol/L)	135-145	137	133	135	135
Serum amylase (U/L)	30-110	340	-	61	-
FT3(pmol/L)	3.28-6.47	6.08	7.04	7.4	6.78
FT4(pmol/L)	7.64-16.03	8.35	8.75	10.23	12.5
TSH(mIU/L)	0.56-5.91	3.31	7.524	5.718	3.916
lactic acid (mmol/L)	1-1.7	4.5	-	6.63	-
ICA	-	0.05	-	-	-
IAA	-	0.05	-	-	-
GAD-Ab(Iu/ml)	0-10	0.56	-	-	-
IA-2A(Iu/ml)	＜10	＜0.7	-	-	-
Znt8(Au/ml)	＜10	2.63	-	-	-

BMI: body mass index; Hb: Hemoglobin; ALT: alanine aminotransferase; AST: aspartate aminotransferase; TC: total cholesterol; TG: triglyceride; LDL: low-density lipoprotein cholesterol; FPG: fasting plasma glucose; Cr: creatinine; Ua: uric acid; UACR: urine albumin-to-creatinine ratio; K^+: serum potassium; Na^+： serum sodium; FT3: Free Triiodothyronine; FT4: free thyroxine; TSH: Thyroid-stimulating hormone; ICA: islet cell autoantibody; IAA: insulin autoantibodies; GAD-Ab: antibody against glutamate decarboxglase; IA-2A: tyrosine phosphatase-like IA-2 autoantibody; Znt8: Zinc transporter 8 antibody.
